# miR-124-3p downregulates EGR1 to suppress ischemia-hypoxia reperfusion injury in human iPS cell-derived cardiomyocytes

**DOI:** 10.1038/s41598-024-65373-x

**Published:** 2024-06-27

**Authors:** Qiaoke Yang, Kozue Murata, Tadashi Ikeda, Kenji Minatoya, Hidetoshi Masumoto

**Affiliations:** 1 Department of Cardiovascular Surgery, Graduate School of Medicine, Kyoto University, 54 Kawara-cho, Shogoin, Sakyo-ku, Kyoto, 606-8507 Japan; 2 Clinical Translational Research Program, RIKEN Center for Biosystems Dynamics Research, 2-2-3 Minatojima Minamimachi, Chuo-ku, Kobe, 650-0047 Japan

**Keywords:** Stem cells, Cardiology, Medical research

## Abstract

Ischemic heart diseases are a major global cause of death, and despite timely revascularization, heart failure due to ischemia-hypoxia reperfusion (IH/R) injury remains a concern. The study focused on the role of Early Growth Response 1 (EGR1) in IH/R-induced apoptosis in human cardiomyocytes (CMs). Human induced pluripotent stem cell (hiPSC)-derived CMs were cultured under IH/R conditions, revealing higher EGR1 expression in the IH/R group through quantitative real-time polymerase chain reaction (qRT-PCR) and Western blotting (WB). Immunofluorescence analysis (IFA) showed an increased ratio of cleaved Caspase-3-positive apoptotic cells in the IH/R group. Using siRNA for EGR1 successfully downregulated EGR1, suppressing cleaved Caspase-3-positive apoptotic cell ratio. Bioinformatic analysis indicated that EGR1 is a plausible target of miR-124-3p under IH/R conditions. The miR-124-3p mimic, predicted to antagonize EGR1 mRNA, downregulated EGR1 under IH/R conditions in qRT-PCR and WB, as confirmed by IFA. The suppression of EGR1 by the miR-124-3p mimic subsequently reduced CM apoptosis. The study suggests that treatment with miR-124-3p targeting EGR1 could be a potential novel therapeutic approach for cardioprotection in ischemic heart diseases in the future.

## Introduction

The global prevalence of cardiovascular diseases is on the rise, with ischemic heart diseases such as acute myocardial infarction (AMI) being significant contributors to morbidity and mortality worldwide^1^. While timely revascularization is performed following the onset of AMI to limit the size of the infarct and subsequent ventricular remodeling^2^, some cases may result in cardiac dysfunction and heart failure, possibly due to ischemia-hypoxia reperfusion (IH/R) injury following revascularization^3^. Extensive research has been conducted to elucidate the molecular mechanisms of IH/R-mediated apoptosis and to mitigate reperfusion injury by reducing myocardial cell death^4^. Previous studies involving rat or mice cardiomyocytes (CMs) have found that early growth response 1 (EGR1), a prototypic Cys2-His2 type zinc finger transcription factor, plays a role in triggering ischemia–reperfusion injury and is rapidly induced by various stimuli such as growth factors, pro-inflammatory cytokines, and hypoxia^5,6^. However, there is currently no evidence to suggest that EGR1 is involved in exacerbating IH/R-induced apoptosis in human CMs.

The use of human induced pluripotent stem cells (hiPSCs) has garnered significant attention in cardiovascular medical research^7,8^. hiPSCs are reprogrammed human cells that possess characteristics similar to embryonic stem cells, including the ability to self-renew and differentiate into various types of somatic cells, including CMs^9,10^. The hiPSC-derived CM (hiPSC-CM) model has been employed to study cardiac ischemia–reperfusion injury, allowing us to replicate cell morphology, expression of structural proteins, beating frequency, depolarization time, field potential duration, and conduction velocity during different stages of the ischemia–reperfusion event^11^. In the long term, the clinical application of hiPSC-derived CMs holds great potential as a powerful approach for modeling cardiovascular diseases.

MicroRNAs (miRNAs or miRs) are endogenous non-coding RNAs consisting of 21–25 nucleotides. Their primary function is to facilitate post-transcriptional regulation of target genes by binding to the 3' untranslated region (UTR) of messenger RNAs (mRNAs)^12,13^. It has been reported that miRNAs are involved in the regulation of at least 30% of human genes^14^. miRNAs play a crucial role in cell differentiation, proliferation, and survival and are known to be dysregulated in various diseases, including cancer, hepatitis, and cardiovascular diseases. The ability of miRNAs to target multiple mRNAs that are altered in disease conditions makes them potential candidates for therapeutic interventions^15^. miR-124-3p, a mature form of miR-124, has been implicated in various human diseases, such as cancer^16,17^, by downregulating the expression of EGR1 in human endothelial cells^18^ and rat hippocampal neurons^19^, which may modulate cell autophagy, apoptosis, and disease progression^20^. Furthermore, miR-124-3p has been identified as a potential regulator in AMI according to human clinical data^21^, which motivated our investigation into the therapeutic potential of miR-124-3p in the context of IH/R injury in the human heart by downregulating EGR1.

In our current research, we have confirmed that EGR1 is upregulated in hiPSC-CMs under conditions simulating IH/R and functions as a regulator of IH/R-induced apoptosis. Through the use of siRNA to silence the *EGR1* gene, we were able to suppress the apoptosis promoted by IH/R. Additionally, miR-124-3p has been identified as an inhibitor of EGR1. Overexpression of miR-124-3p in hiPSC-CMs resulted in a lower rate of apoptosis in the IH/R group compared to the control groups. These findings may offer new targets and approaches for cardio-protection against IH/R conditions.

## Results

### IH/R enhances EGR1 expression and promotes apoptosis in hiPSC-CMs

**Figure 1:**
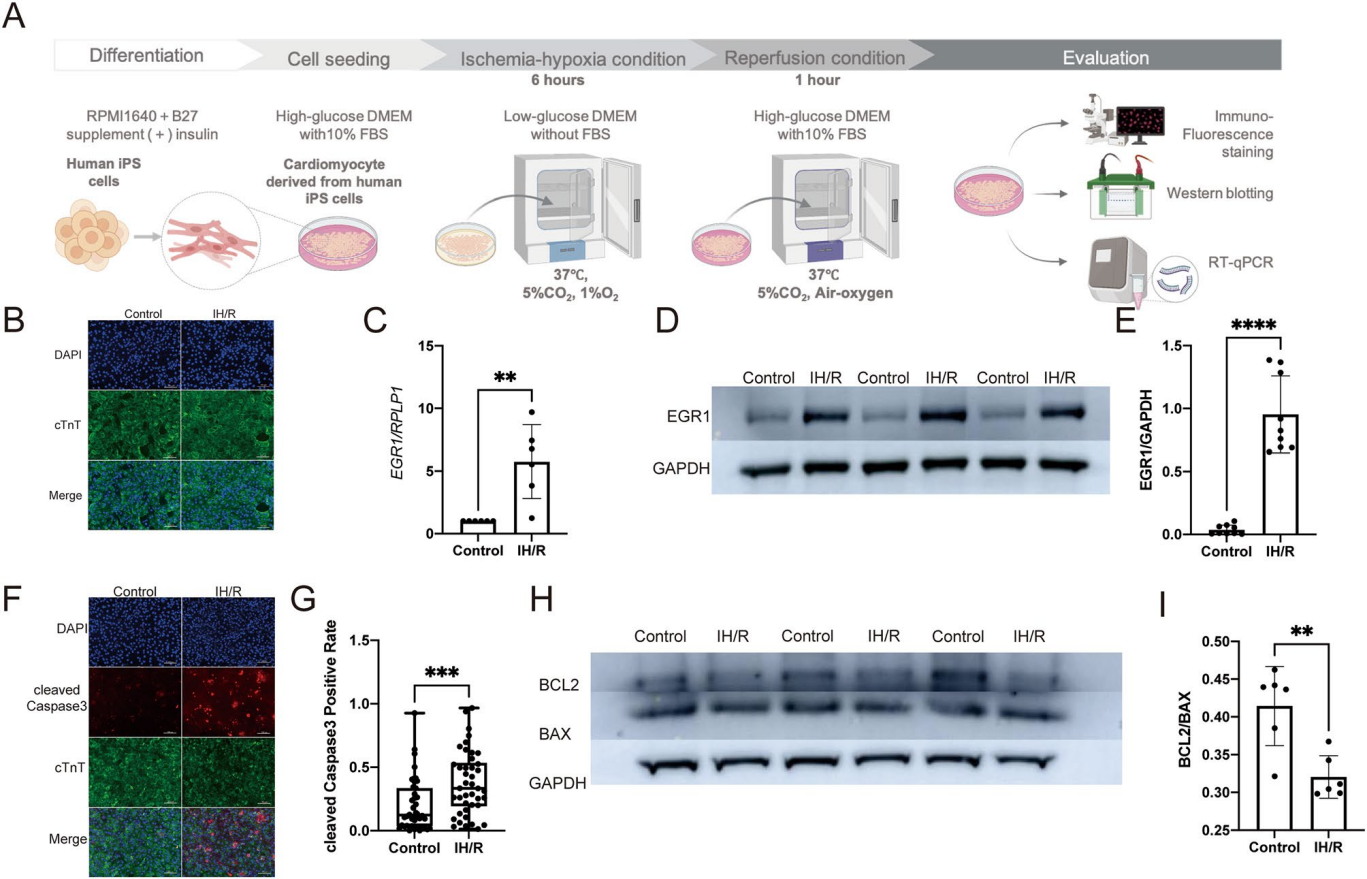
IH/R increases the expression of EGR1 and induces apoptosis in hiPSC-CMs. (**A**) A chart of the experiments of the induction of IH/R in hiPSC-CMs. (**B**) Representative immunofluorescence staining for cardiac isoform of troponin-T (cTnT) for hiPSC-CMs. (**C**) qRT-PCR of human *EGR1* and *RPLP1* (endogenous control) for hiPSC-CMs (n = 6). **P < 0.01. (**D**,**E**). Western blotting of human EGR1 and GAPDH (endogenous control) (n = 9). ****P < 0.0001. (**F**,**G**). Immunofluorescence staining for cleaved Caspase-3 (red), cTnT (green) and DAPI (blue) (n = 42). Scale bars 100 μm. ***P < 0.001. (**H**). Western blotting of human BCL2, BAX and GAPDH (endogenous control) (n = 6). (**I**). The ratio of BCL2/BAX in Control group and IH/R group, **P < 0.01.

To investigate the involvement of EGR1 in human CMs, and to examine whether IH/R treatment triggers apoptosis in human CMs via EGR1 activation, we exposed hiPSC-CMs to IH/R conditions (Fig. 1A,B). Quantitative RT-PCR analysis (Fig. 1C) and Western blotting (Fig. 1D,E; Supplementary Fig. S1) revealed a significant upregulation of EGR1 expression under IH/R conditions. These findings confirm that EGR1 expression is enhanced under IH/R conditions in hiPSC-CMs. Immunofluorescence staining for cleaved Caspase-3 showed a significantly higher rate of apoptosis in the IH/R group compared to the control group (Fig. 1F,G). Additionally, the BCL2 to BAX ratio was used as an indicator of apoptosis extent. Western blotting results indicated a significantly lower BCL2/BAX ratio in the IH/R group compared to the control group (Fig. 1H,I; Supplementary Fig. S1). These findings indicate that apoptosis of hiPSC-CMs is promoted under IH/R conditions.

### Downregulation of EGR1 expression suppresses IH/R-mediated apoptosis in hiPSC-CMs

**Figure 2:**
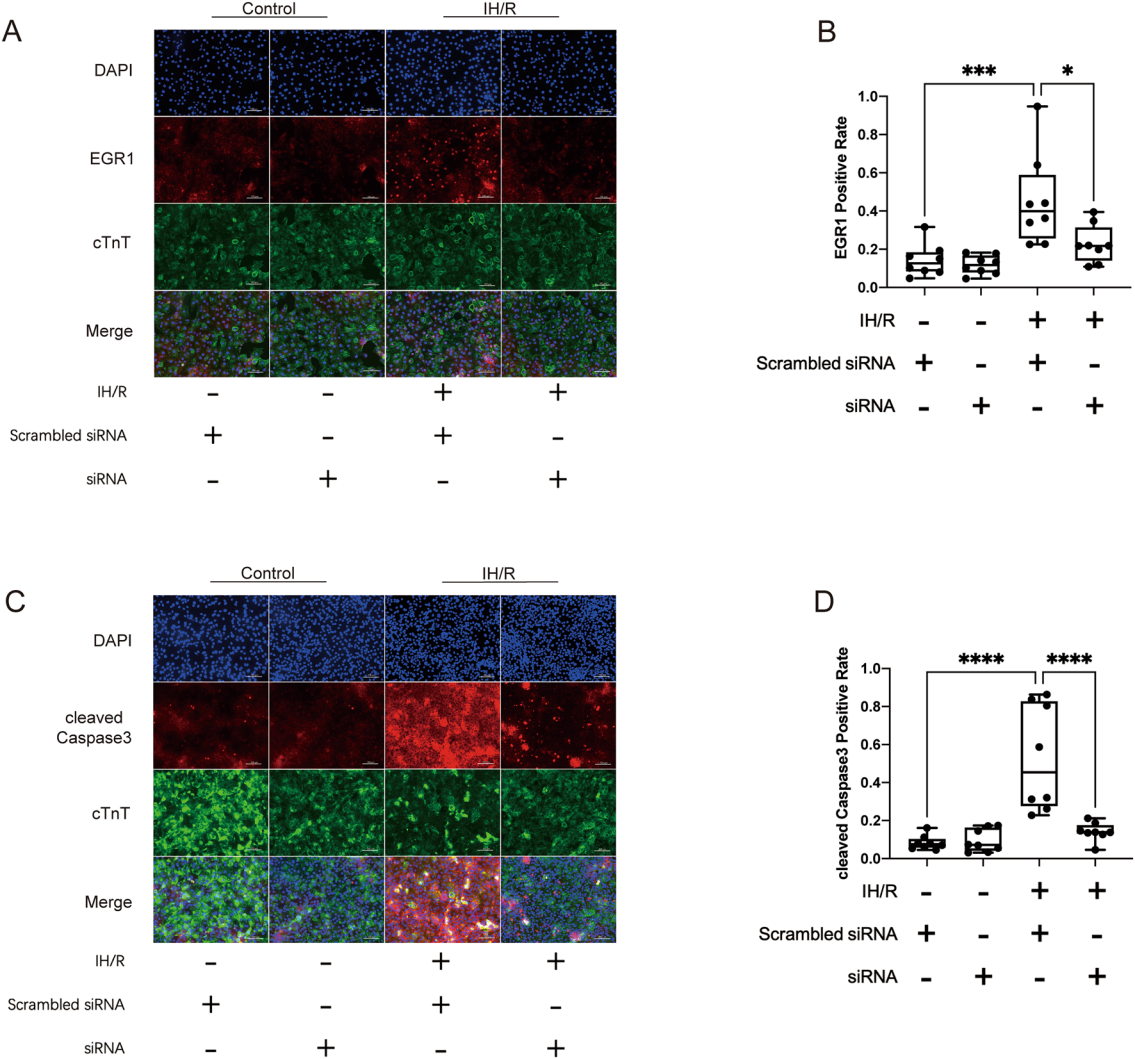
The suppression of apoptosis mediated by IH/R in hiPSC-CMs is achieved by downregulating EGR1 using siRNA. (**A**,**B**) Immunofluorescence staining for EGR1 (red), cTnT (green) and DAPI (blue) (n = 8). Scale bars 100 μm. ***P < 0.001, *P < 0.05. (**C**,**D**) Immunofluorescence staining for cleaved Caspase-3 (red), cTnT (green) and DAPI (blue) (n = 8). Scale bars 100 μm. ****P < 0.0001, ****P < 0.0001.

Next, our aim was to suppress EGR1 expression using RNA interference and examine whether inhibiting EGR1 would attenuate apoptosis in hiPSC-CMs. We designed a small interfering RNA (siRNA) targeting EGR1 mRNA and transfected it, along with scrambled siRNA, into hiPSC-CMs. The results of IFA showed that IH/R treatment increased the expression of EGR1. Furthermore, the siRNA successfully decreased the expression of EGR1 under IH/R conditions (Fig. 2A,B). To investigate apoptosis, IFA for cleaved Caspase-3 demonstrated that IH/R treatment promoted CM apoptosis. Importantly, the siRNA targeting EGR1 inhibited the apoptosis promoted by IH/R (Fig. 2C,D).

### Prediction of the target of miR-124-3p through bioinformatic analyses

**Figure 3:**
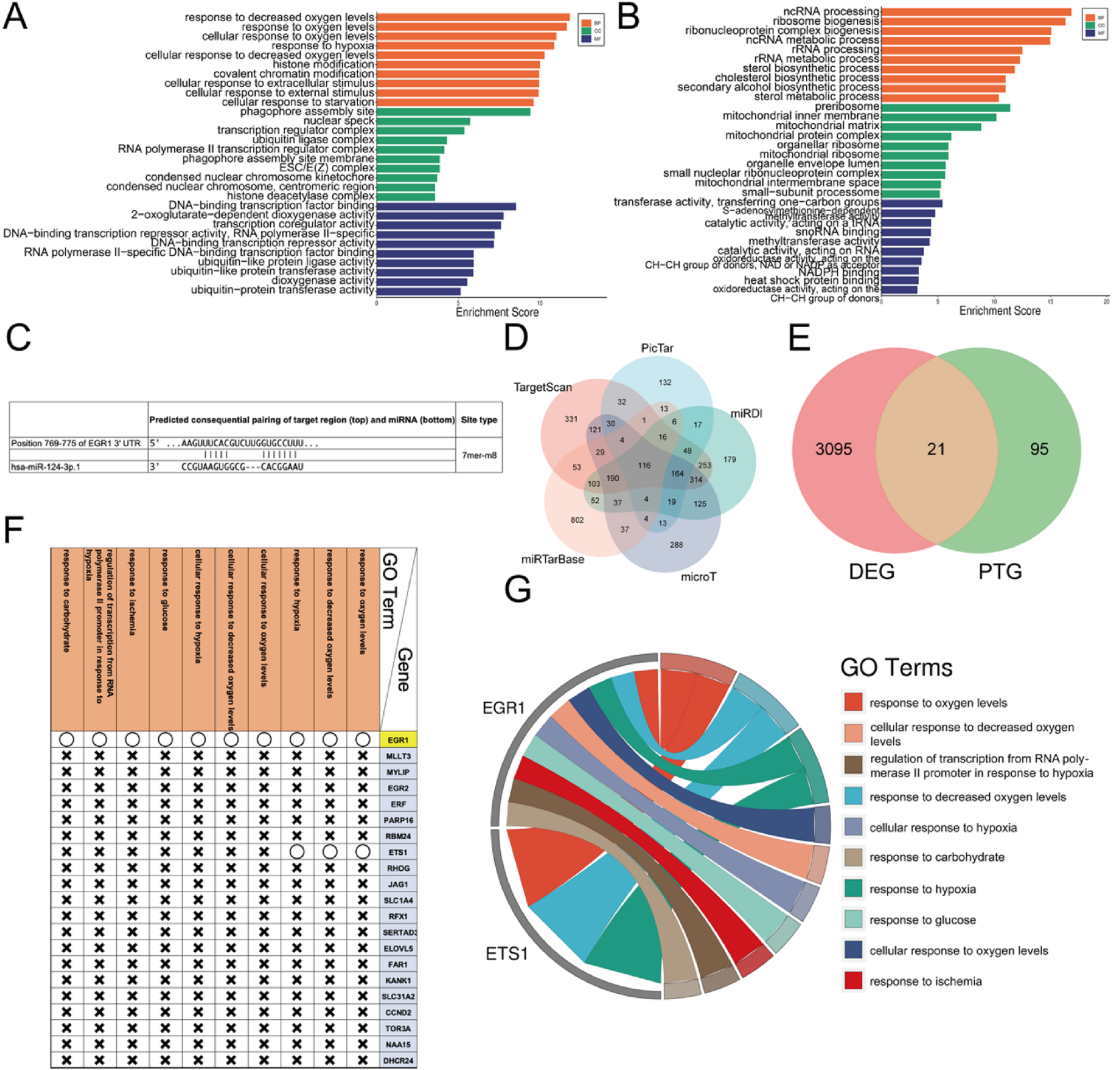
Prediction of the target of miR-124-3p. (**A**) GO analysis of 1426 upregulated genes. BP: biological process, CC: cellular component, MF: molecular function. (**B**) GO analysis of 1690 downregulated genes. BP: biological process, CC: cellular component, MF: molecular function. (**C**) The matching sequence of human EGR1 mRNA 3' UTR and human miR-124-3p analyzed by TargetScan. (**D**) The intersection of the prediction results for the target of miR-124-3p from 5 database. (**E**) The intersection of 116 Predicted Target Gene (PTG) and the 3116 Differential Expression Gene (DEG). (**F**) The involvement condition of the 21 candidate genes with the 10 BPs related to IH/R. (**G**) The chordal diagram of the involvement condition of the 21 candidate genes with the 10 BPs related to IH/R.

Previous studies have indicated that EGR1 is targeted by miR-124-3p^18,19^. To elucidate the molecular pathway regulating miR-124-3p under IH/R conditions, we employed various bioinformatic tools for miRNA target prediction to investigate potential binding sites of miR-124-3p. Firstly, we conducted GO analysis on both upregulated and downregulated genes. The analysis of all 1426 upregulated genes indicated an increase in biological processes (BPs) related to IH/R, such as the response to hypoxia and cellular response to starvation (Fig. 3A). Similarly, the analysis of all 1690 downregulated genes highlighted crucial BPs for cardiac myocytes, including the sterol biosynthetic process, cholesterol biosynthetic process, and sterol metabolic process, within the top 10 most significantly suppressed BPs (Fig. 3B). Notably, the TargetScan database predicted a potential binding site for miR-124-3p at positions 769–775 within the EGR1 3’ UTR (Fig. 3C). Through an examination across five different databases, we identified 116 candidate target genes for miR-124-3p (Fig. 3D). By comparing these 116 Predicted Target Genes (PTGs) with the 3116 Differentially Expressed Genes (DEGs; 1426 upregulated and 1690 downregulated), we selected 21 genes as potential targets of miR-124-3p under IH/R conditions (Fig. 3E). The 21 genes are presented in Fig. 3F. Remarkably, among these 21 genes, EGR1 stands out as the sole gene involved in all 10 BPs related to IH/R-associated apoptosis (Fig. 3F,G). These findings collectively suggest that EGR1 is a plausible target of miR-124-3p under IH/R conditions.

### miR-124-3p attenuates IH/R-mediated apoptosis of hiPSC-CMs by downregulating EGR1

To downregulate human EGR1 in human CMs, we used miR-124-3p, which was confirmed to be effective based on the results presented in Fig. 3. In our experimental setup, we transfected hiPSC-CMs with miR-124-3p mimic, negative miRNA, and miRNA positive control (designed to target GAPDH as a measure of transfection efficiency). Following transfection, the cells underwent IH/R treatment.

**Figure 4:**
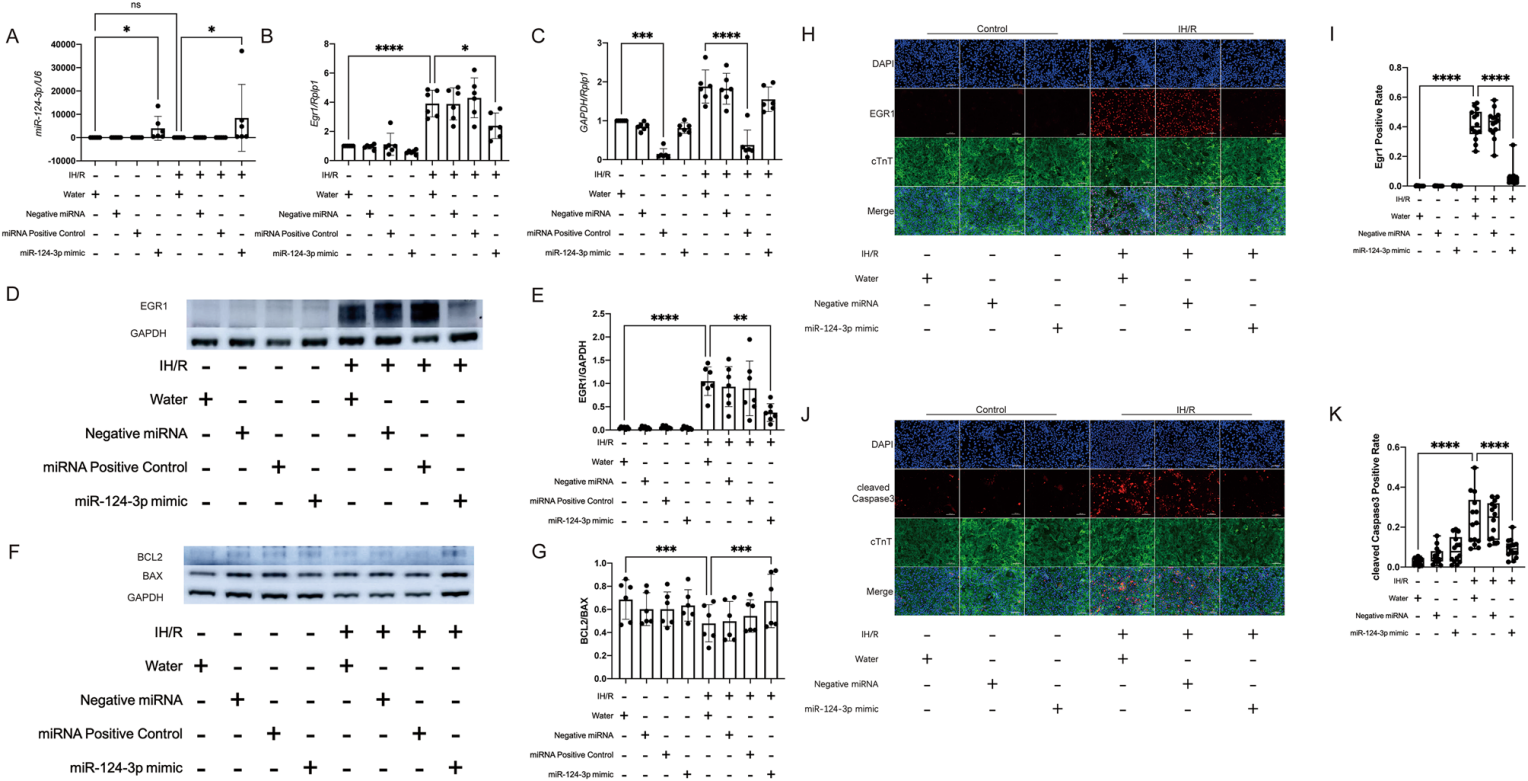
miR-124-3p inhibits the pro-apoptotic effect of IH/R on hiPSC-CMs by downregulating EGR1. (**A**) qRT-PCR of human miR-124-3p and U6 (endogenous control) (n = 6). *P < 0.05. (**B**) qRT-PCR of *EGR1* and *RPLP1* (endogenous control) (n = 6). ****P < 0.0001, *P < 0.05. (**C**) qRT-PCR of *GAPDH* and *RPLP1* (endogenous control) (n = 6). This experiment was conducted to demonstrate the technical efficiency of miRNA transfection. miRNA positive control was designed to antagonize GAPDH mRNA. ***P < 0.001, ****P < 0.0001, (**D**,**E**) Western blotting of EGR1 and GAPDH (endogenous control) (n = 7). **P < 0.01, ****P < 0.0001. (**F**). Western blotting of BCL2, BAX and GAPDH (endogenous control) (n = 6). (**G**). The ratio of BCL2/BAX in Control and IH/R groups. ***P < 0.001, ***P < 0.001 (**H**,**I**) Immunofluorescence staining for EGR1 (red), cTnT (green) and DAPI (blue) (n = 14). Scale bars 100 μm, ****P < 0.0001. (**J**,**K**) Immunofluorescence staining for cleaved Caspase-3 (red), cTnT (green) and DAPI (blue) (n = 14). Scale bars 100 μm, ****P < 0.0001.

qPCR analysis of miR-124-3p demonstrated that the miR-124-3p mimic effectively increased the expression of miR-124-3p in the cells (Fig. 4A). The Introduction of the miR-124-3p mimic significantly decreased EGR1 expression in the IH/R group (Fig. 4B). The qPCR analysis of GAPDH, serving as the miRNA positive control, demonstrated effective downregulation of GAPDH expression in both IH/R and control conditions, confirming successful transfection (Fig. 4C). These findings were consistent with the Western blot analysis of EGR1, which showed similar trends (Fig. 4D,E; Supplementary Fig. S2). Western blotting of BCL2 and BAX were also utilized in the assessment of apoptosis. The ratio of BLC2/BAX demonstrated that IH/R induced apoptosis significantly. Meanwhile, treatment with the miR-124-3p mimic effectively increased the BCL2/BAX ratio, indicating the miR-124-3p suppressed the apoptosis induced by IH/R. (Fig. 4F,G; Supplementary Fig. S2).

The IFA results showed that IH/R treatment significantly increased the expression of EGR1 compared to the control group. However, with the introduction of miR-124-3p, there was an effective decrease in the expression of EGR1 in the IH/R condition (Fig. 4H,I). The IFA results for cleaved Caspase-3 indicated that the IH/R treatment induced apoptosis in CMs. Conversely, the introduction of miR-124-3p inhibited the apoptosis promoted by IH/R (Fig. 4J,K).

## Discussion

Myocardial reperfusion injury was first proposed by Jennings et al. in 1960^22^. The damage to myocardial tissue caused by reperfusion can be more extensive than the damage caused by the original ischemic insult, and it may account for up to 50% of the final size of the myocardial infarction^23^. Reactive oxygen species (ROS) and oxidative stress (OS) are considered to be crucial mediators in reperfusion injury^24^. During the reperfusion process, ROS levels increase due to various factors such as increased formation of xanthine oxidase, neutrophil respiratory burst, and damage to the mitochondrial electron transport chain^25^. Especially during the early phase of reperfusion, excessive amounts of ROS are produced, which can directly damage the myocardium and indirectly activate intracellular signaling pathways, leading to further injury^26^. The inflammatory response, often accompanied by OS, involves the infiltration of inflammatory cells into the myocardial infarction zone, leading to elevated levels of ROS, release of cytokines, and activation of apoptotic and necrotic death pathways^27^. It has also been reported that mitochondrial calcium overload^28^ and energy metabolism disorders^29^ are involved in the progression of cardiac tissue damage during IH/R. To attenuate the progression of myocardial injury caused by IH/R, it is essential to identify key molecules that primarily contribute to this process in CMs. Downregulating specific culprit molecules in human CMs may potentially serve as a new strategy for treating ischemic heart diseases.

EGR1 is a member of the EGR family and is known by various names including NEFI-A, Zif268, Krox-24, and TIS85^30^. EGR1 possesses an activation regulatory region, a repressive regulatory region, and three Cys2-His2 subclass zinc finger structures. These structures specifically recognize and bind target genes, thereby regulating transcription^30^. EGR1 is widely expressed in different cell types and plays a role in crucial physiological processes such as cell proliferation, differentiation, invasion, and apoptosis^31^. Studies have also reported the induction and activation of EGR1 in the early stages of hypoxia/ischemia. Following reperfusion, EGR1 triggers the expression of immune effectors and inflammatory effectors^32^. In our current research, we conducted RNA-seq analysis and confirmed that EGR1 is a critical factor in IH/R-induced apoptosis of hiPSC-CMs. Additionally, through siRNA administration, we demonstrated that downregulating EGR1 reduced CM apoptosis in the IH/R condition, further suggesting the involvement of EGR1 in this process. Furthermore, we presented evidence for the therapeutic potential of miR-124-3p in antagonizing the mRNA of EGR1. This finding may open up new possibilities for the development of a novel therapeutic approach targeting EGR1.

Previous studies have demonstrated the involvement of miRNAs in the regulation of EGR1 in cardiovascular disorders such as heart failure and myocardial infarction^20^. In recent years, several miRNA databases based on bioinformatics and computer science, such as miRBase, TargetScan, DIANA-microT-CDS, miRwalk, miRDB, and micro-TarBase, have been developed^33,34^. These databases enable the prediction of complementary binding sites between human miRNAs and mRNAs, providing valuable tools for selecting miRNAs that target specific genes. There is a previous study indicating that miR-145 downregulates EGR1 in mouse vascular smooth muscle cells^35^. Considering that the apoptosis of rat^5^ and mouse^6^ CMs is reported to be regulated by EGR1, and the sequences of *Egr1* mRNA and miR-145 are consistently preserved in mouse and rat, it might be speculated that miR-145 can downregulate EGR1 and subsequently attenuate CM apoptosis in both mouse and rat. To investigate whether miR-145 may have a similar effect in human CMs, we referred to the TargetScan database. Interestingly, while miR-145 targets *Egr1* mRNA in both rats and mice, we discovered that human miR-145 does not target human *EGR1* mRNA. We utilized several miRNA databases again (Fig. 3) and identified the potential use of miR-124-3p in downregulating EGR1. Through experimental validation, we have shown that miR-124-3p can downregulate human EGR1 and inhibit IH/R-mediated apoptosis in hiPSC-CMs. miR-124-3p has also been reported to have inhibitory effects on the proliferation and invasion of breast cancer^16^ and pancreatic ductal adenocarcinoma^17^. The diverse biological activities of miR-124-3p also raise concerns regarding its potential unexpected effects when used for the treatment of ischemic heart diseases. To mitigate these unforeseen effects, the development of a drug delivery system becomes crucial for the future clinical implementation of the current strategy^36^.

Small animal models, especially mice, have provided significant insights into the molecular and cellular mechanisms of cardiovascular biology. However, there are notable differences in cardiac characteristics between mice and humans, such as heart rate, oxygen consumption, adrenergic receptor ratios, and response to the loss of regulatory proteins^37^. Therefore, human-based models are crucial for cardiovascular research. In recent years, iPSC technology has emerged as a valuable tool for modeling heart disease, drug screening, and testing for cardiotoxicity^38^. These efforts aim to utilize iPSCs to replicate human cardiac conditions and provide a more accurate platform for studying cardiovascular diseases and developing potential therapeutic interventions. In our present study, we specifically employed hiPSC-CMs as our experimental material. By using hiPSC-CMs, we aimed to obtain more precise and promising results that could potentially be applied in future clinical settings.

siRNA is artificially designed to perfectly complement and pair with the target gene. In contrast, miRNA is known to exhibit a flexible complementarity range of 20–90% with the target gene^39^. This flexibility in targeting may potentially lead to toxicity in normal cells^40^. Additionally, the effectiveness of miRNA can vary depending on the cellular environment, meaning that the same miRNA may have different targets in different cell types, resulting in unexpected effects^41^. However, miRNA offers its own advantages compared to siRNA. The most significant advantage is that miRNAs can target multiple coding genes or non-coding genes. In diseases involving multiple genes, a single miRNA may regulate specific disease-related pathways. For example, it has been reported that miRNAs targeting genes implicated in multiple pathways can be used to achieve broad silencing of pro-tumoral pathways^41^. Furthermore, miRNA has been found to exhibit diverse biological activities beyond simply suppressing the expression of specific genes. Previous research indicates that miRNAs can recruit protein complexes to the AU-rich elements of mRNA or derepress mRNA translation by interacting with translation-blocking proteins^42^. Moreover, miRNAs can globally influence protein synthesis by enhancing ribosome biogenesis^43^ or switching the regulation from repression to activation of target gene translation under cell cycle arrest conditions^42^. While miRNA therapy is still in its early clinical stages, further development is anticipated for broader clinical applications.

In the realm of cardiovascular disease treatment, both miRNA and siRNA-based drugs are undergoing clinical trials. For instance, CDR132L, an anti-miR targeting miR-132, has been proven to be safe and effective in heart failure patients^44^. On the other hand, there are also several siRNA drugs in testing phases. Inclisiran, an siRNA directed at PCSK9, has shown significant reductions in low-density lipoprotein cholesterol levels and good tolerability in severe cases of hypercholesterolemia^45^. Similarly, AMG890 (also known as Olpasiran), a siRNA compound designed to reduce apo(a) synthesis, has demonstrated significant decreases in lipoprotein(a) concentrations in patients with atherosclerotic cardiovascular diseases in recent clinical trials^46^. Further exploration of the therapeutic potentials of miRNA and siRNA in cardiovascular diseases is warranted.

Despite timely reperfusion, 25% of acute myocardial infarction survivors develops chronic heart failure due to the spectrum of reperfusion-associated pathologies^3^. Research has been focused on therapeutic agents that would render myocardial cells more resistant to the deleterious effects of ischemia and reperfusion, a concept known as “cardioprotection,” which involves manipulating cellular events during ischemia and reperfusion to reduce myocardial cell death^3^. For example, the selective κ-opioid agonist U50,488H has been shown to be protective against myocardial ischemia–reperfusion injury^47^. In the present study, we have identified a new approach to cardioprotection, in which EGR1, a crucial factor accelerating IH/R-mediated CM apoptosis, can be downregulated.

This study has several limitations. Firstly, the conditions regarding IH/R were not thoroughly investigated. While the experimental conditions of IH/R adopted in this study allowed us to induce cell death and further demonstrate the inhibitory effect of miR-124-3p on cell death, conducting preliminary experiments to identify optimal IH/R conditions based on the extent of cell death would have been necessary for a more rigorous examination. Secondly, in our experiments investigating the suppression of EGR1 expression using siRNA, including the design of siRNA, we were not able to sufficiently explore the therapeutic effects of EGR1 suppression by siRNA. The types of siRNA used in this study were limited, and the use of other sequences of siRNA or a combination of siRNAs may achieve a better effect of EGR1 downregulation. As discussed in the discussion section, there is potential for utilizing siRNA-based therapy in IH/R, but since this study primarily focused on the therapeutic effects of miRNA, the experimentation with siRNA was somewhat inadequate. Further investigation is required to establish optimal treatment strategies based on gene expression regulation for inhibiting IH/R-mediated injury in myocardial cells. Thirdly, we have demonstrated the effects of IH/R and the therapeutic effects of miR-124-3p in human cardiac myocytes using hiPSC-CMs in this study. However, we believe that if we could demonstrate similar findings in primary cultures of CMs derived from human cardiac tissue, it would further expand the potential clinical applications of this therapeutic strategy. Unfortunately, due to difficulties in obtaining experimental materials and a lack of expertise in conducting primary culture of CMs from human cardiac tissue in this study, we were unable to carry out this experiment. Nonetheless, we believe it is essential to conduct experiments using primary culture in the future to demonstrate that similar mechanisms also operate in cardiac myocytes derived from live human cardiac tissue.

## Methods

All methods were performed in accordance with the relevant guidelines and regulations (Declaration of Helsinki).

### Cell culture

The hiPSC-CMs were prepared following a previously reported protocol^48^ with some modifications. Initially, hiPSCs (201B6 line)^49^ were expanded and cultured using StemFit AK02N medium (AJINOMOTO, Tokyo, Japan). Upon reaching confluence, the cells were dissociated using TrypLE Select (Thermo Fisher) and then resuspended in a mixture of 0.5 mM ethylenediaminetetraacetic acid in phosphate-buffered saline (PBS) (1:1). Subsequently, the cells were passaged at a density of 5000–8000 cells/cm^2^ every 5–7 days in AK02N medium containing iMatrix-511 silk (0.125 µg/cm^2^), a laminin E8 fragment (FUJIFILM Wako Pure Chemical Corp., Osaka, Japan), and a ROCK inhibitor Y-27632 (10 µM) (FUJIFILM Wako). For cardiovascular cell differentiation, dissociated iPSCs were seeded onto Matrigel (Corning)-coated plates (1:60 dilution) at a density of 300,000–400,000 cells/cm^2^ in AK02N medium supplemented with Y-27632 (10 µM). When the cells reached confluence, Matrigel (1:60 dilution in AK02N) was applied one day before initiating differentiation. At this point (differentiation day 0; d0), the AK02N medium was replaced with RPMI + B27 Insulin (−) medium containing 100 ng/mL Activin A (R&D, Minneapolis, MN, USA), and 5 µM CHIR99021 (Tocris Bioscience, Bristol, UK). After 24 h, the medium was supplemented with 10 ng/mL bone morphogenetic protein 4 (BMP4) (R&D) and 10 ng/mL basic fibroblast growth factor (bFGF) (FUJIFILM Wako) on d1 and maintained for 4 days without changing the culture medium. On d5, the culture medium was switched to RPMI1640 + B27 Insulin (+) medium containing 2.5 µM IWP4 (Stemgent, Cambridge, MA, USA), and 5 µM XAV939 (Merck, Kenilworth, NJ, USA). The culture medium was refreshed with RPMI1640 + B27 Insulin (+) medium every other day. Beating cells started to appear at d11 to d13, indicating successful differentiation into cardiomyocytes. The cultured cells were then transferred from RIKEN, Kobe, Japan, to Kyoto University, Kyoto, Japan, using the iP-TEC live cell transportation system (SANPLATEC CO., Ltd., Osaka, Japan) within 2 h. For dissociation, the CMs were treated with Accumax (STEMCELL Technologies, Vancouver, Canada) and then cultured with attachment medium containing alpha minimum essential medium (αMEM) from Thermo Fisher, supplemented with 10% FBS, 50 units/mL of Penicillin–Streptomycin (Thermo Fisher) and 5 × 10^−5^ M of 2-mercaptoethanol (Thermo Fisher). The cells were seeded at 10 × 10^4^/cm^2^ density on a 6-well plate (FALCON, Corning, USA) or 12-well plate (FALCON, Corning, USA) coated with FBS (Biowest, Nuaillé, France). The experiments were conducted 1 day after the cell seeding.

### Ischemia and hypoxia-reperfusion (IH/R) culture

The cells were cultured in a MINI cell Benchtop CO_2_ incubator (WAKENBTECH CO. LTD, Kyoto, Japan) under specific gas conditions to simulate various stages of ischemia and reperfusion. To create a hypoxic environment, the cells were exposed to 1% O_2_ and 5% CO_2_. For the ischemic condition, a low-glucose (1.0 g/L) DMEM (Thermo Fisher Scientific) without 10% FBS was used. Subsequently, the medium was changed back to fresh high-glucose (4.5 g/L) DMEM with 10% FBS to mimic reperfusion. The cells were also transferred to an incubator with normal room air oxygen concentration and 5% CO_2_ during this reperfusion phase. To induce conditions for ischemia/hypoxia and reperfusion, the cells were subjected to ischemic and hypoxic conditions for 6 h. Afterward, they underwent reperfusion for 1 h.

### Transfection of small interfering RNAs (siRNAs) and microRNAs (miRNAs)

HiPSC-CMs were utilized for the transfection of siRNAs and miRNA mimics. The transfection process involved using Lipofectamine RNAiMAX Reagent (Thermo Fisher). The siRNAs were synthesized by Sigma-Aldrich Co. (St. Louis, MO, USA). As a Negative Control (NC) siRNA, scrambled siRNA obtained from QIAGEN (Venlo, The Netherlands) was used. Before reseeding the cells, 12-well plates (for siRNA experiments) and 6-well plates (for miRNA experiments) were coated with FBS overnight. For the siRNA experiments, the hiPSC-CMs were divided into 6 groups as follows: IH/R siRNA group, IH/R Scrambled siRNA group, IH/R no-treatment group, NC siRNA group, NC Scrambled siRNA group, and NC no-treatment group. After reseeding, the CMs were cultured overnight. Then, each group was treated with Lipofectamine along with the specific siRNAs for 6 h, followed by a change to fresh culturing medium and continued culturing for 48 h. After this, the four IH/R groups underwent IH/R treatment, while the four NC groups were cultured under normal conditions for the same duration. Regarding the miRNA experiments, the miR-124-3p mimic used was purchased as a product listed in miRCURY LNA miRNA Mimic (QIAGEN). Additionally, negative and positive miRNA control were obtained from BIONEER (Daejeon, Korea). The transfection protocol for miRNAs was the same as that used for siRNAs. The siRNA was transfected in a final concentration of 0.1 μM, while the miRNA was transfected in a final concentration of 5 nM, According to the product specification. The sequences of the siRNAs and miRNAs are provided below.

**Table:** 

siEGR1	AGAGGCAUACCAAGAUCCA[dT][dT] (Rv: UGGAUCUUGGUAUGCCUCU[dT][dT])
Scrambled siRNA	UUCUCCGAACGUGUCACGU[dT][dT] (Rv: ACGUGACACGUUCGGAGAA[dT][dT])
Hsa-mir-124-3p	UAAGGCACGCGGUGAAUGCCAA

### Quantitative real-time polymerase chain reaction (qRT-PCR)

The hiPSC-CM cells were collected using Accumax (STEMCELL Technologies, Vancouver, Canada). After 15 min of incubation, it was neutralized by adding 5 times the volume of culture medium. Cell pellets were collected after centrifugation at 3000 rpm for 5 min. Total RNA was extracted using the RNeasy Mini Kit (QIAGEN). Subsequently, reverse transcription to cDNA was performed using the QuantiTect Reverse Transcription Kit (QIAGEN). qRT-PCR was carried out using the StepOnePlus real-time PCR system (Thermo Fisher). The reaction mixture was prepared in a 96-well plate, consisting of 10 μl of THUNDERBIRD SYBR qPCR Mix (TOYOBO, Osaka, Japan), 0.4 μl of 50 × ROX reference dye (TOYOBO), 2 μl of cDNA (diluted 5 times), and 6.1 μl of RNase-free water (QIAGEN) for each well. The amplification conditions were set as follows: pre-denaturation at 95 °C for 1 min, denaturation at 95 °C for 15 s, annealing at 55 °C for 30 s, extension at 72 °C for 45 s, and 40 cycles. For miRNA analysis, reverse transcription was performed using the miRCURY LNA RT Kit (QIAGEN), and PCR was carried out using the miRCURY LNA SYBR Green PCR Kit (QIAGEN). The amplification conditions were as follows: pre-denaturation at 95 °C for 2 min, denaturation at 95 °C for 10 s, annealing and extension at 56 °C for 60 s, and 40 cycles. The RNA expression level was quantified using the 2^−ΔΔCT^ value. Value of control in each experiment was set as 1, and values in other groups were shown as a fold increase compared to those of control. The primer sequences used in the experiments are provided below.

**Table:** 

Primers	Sequence (5′ → 3′)
hEGR1-Forward	CTGCGACATCTGTGGAAGAAA
hEGR1-Reverse	TGTCTGCTTTCTTGTCCTTCTG
hGAPDH-Forward	GGACTCATGACCACAGTCCA
hGAPDH-Reverse	CCAGTAGAGGCAGGGATGAT
hRPLP1-Forward	AGCCTCATCTGCAATGTAGGG
hRPLP1-Reverse	TCAGACTCCTCGGATTCTTCTTT

### Western blotting

The hiPSC-CM pellets were collected following the procedure outlined in *Quantitative real-time polymerase chain reaction (qRT-PCR)*. To lyse the cells, Sample Buffer (2ME +) (FUJIFILM Wako) was used and heated to 95 °C for 5 min. The resulting samples were then subjected to electrophoresis using a Bolt 8% Bis–Tris Plus gel and diluted Bolt MOPS SDS Running Buffer (20 ×) (Thermo Fisher). After electrophoresis, the gels were cut and transferred to a PVDF membrane using iBolt 2 PVDF Mini Stacks and the iBlot 2 Gel Transfer Device (Life Technologies, Carlsbad, CA, USA). Subsequently, the membrane was blocked using 5% skim milk TBST buffer for 1 h, followed by incubation with the primary antibodies: EGR1 (44D5) Rabbit mAb (#4154, 75 kDa, 1:1000, Cell Signaling Technology, Danvers, MA, USA), BCL2 (D17C4) Rabbit mAb (#3498, 26 kDa, 1:1000, Cell Signaling Technology, Danvers, MA, USA), BAX (E4U1V) Rabbit mAb (#41,162, 20 kDa, 1:1000, Cell Signaling Technology, Danvers, MA, USA) and GAPDH (100,118, 36KD, 1:5000, GeneTex, Irvine, CA, USA) at 4 °C overnight. Afterward, the membranes were washed three times with TBST at room temperature for 10 min each, and then incubated with the Goat anti-Rabbit IgG (H + L) Secondary Antibody HRP (#31,460, 1:10,000, Thermo Fisher) at room temperature for 1 h. Following another three washes with TBST for 10 min each, the images were captured using the ImageQuant LAS 500 (GE, Boston, MA, USA). Finally, the images were quantified using Image J software^50^. As a molecular weight marker, we utilized the PageRuler™ Prestained Protein Ladder (Thermo Fisher) and visualized it using the Colorimetric marker setting on the ImageQuant LAS 500.

### Immunofluorescent imaging

The experiment involved reseeding 100,000 cells into an 8-well chamber slide (Thermo Fisher). After the treatment, the cells were fixed with 4% PFA for 30 min and then permeabilized using 0.5% Tween-20 in PBS for 15 min. To block nonspecific binding, Protein Block (DAKO, Glostrup, Denmark) was applied to the cells for 1 h. Next, the primary antibodies, EGR1 (44D5) Rabbit mAb (#4154), Cleaved Caspase-3 (Asp175) Rabbit mAb (#9661) (Cell Signaling Technology, USA), and Cardiac Troponin T (cTnT) Monoclonal Antibody (13–11) (# MA5-12960, Invitrogen, Thermo Fisher, USA) were diluted 400 times in Antibody Diluent (DAKO) and left to incubate with the cells overnight at 4 °C. After incubation, the cells were washed three times using 0.02% Tween-20 in PBS. Subsequently, four-hundred volume-diluted secondary antibodies, Goat anti-Rabbit IgG (H + L) Cross-Adsorbed Secondary Antibody, Alexa Fluor™ 546 or Goat anti-Mouse IgG (H + L) Cross-Adsorbed Secondary Antibody, Alexa Fluor™ 488 (Invitrogen, Thermo Fisher, USA) were added to the cells and incubated at room temperature for 1 h. Following this, the cells were washed three times with 0.02% Tween-20 in PBS and then sealed with Fluoro-KEEPER Antifade Reagent containing DAPI (Nacalai Tesque, Kyoto, Japan), at room temperature for 30 min. The images were captured and analyzed using the BZ-X810 All-in-One Fluorescence Microscope (KEYENCE, Osaka, Japan). The positive cell ratio was automatically quantified and calculated as the ratio of the nuclei of specific antigen-positive cells over all cell nuclei count. Forty-two random images in each group were used to evaluate CM apoptosis induced by IH/R. Eight random images for each group were analyzed in siRNA experiments, and 14 random images for each group were analyzed in miRNA experiments, respectively.

### RNA-Seq

Total RNA from hiPSC-CMs were collected following the procedure outlined in *Quantitative real-time polymerase chain reaction (qRT-PCR)*. The cDNA library was prepared using the Next GEM Single Cell 39 Gel Bead Kit v3.1 (1,000,129), Chromium Next GEM Chip G Single Cell Kit v3 (PN-1000127), Next GEM Single Cell 39 GME Kit v3.1 (1,000,130), Next GEM Single Cell 39 Library Kit v3.1 (1,000,158), and i7 Multiplex Kit (PN-120262) (10 × Genomics) according to the instructions. Then the cDNA library was run on NextSeq 500 and HiSeq 4000 by Illumina (San Diego, CA, USA). For the quantification of gene expression, an analysis pipeline of ENCODE project (2.3.4) was used (https://www.encodeproject.org/pipelines/ENCPL002LPE/). GRCh38 ENSEMBL release 105 was used for the reference sequence. GRCh38 GENCODE release 39 was used for the gene definition. The values of TPM and FPKM were calculated using STAR-RSEM, a pipeline for analysis (https://github.com/gis-rpd/pipelines/tree/master/rnaseq/star-rsem). For the comparison analysis, normal control (NC) and IH/R samples were compared (N = 1 each). The genes in which Log_2_FC > Log_2_(1.5) were identified as “upregulated” in IH/R group. The genes in which Log2FC < -Log2(1.5) were identified as “downregulated” in IH/R group. The GO analysis were plotted by https://www.bioinformatics.com.cn, an online platform for data analysis and visualization. The predictions of the target of miR-124-3p were analyzed by 5 database: TargetScan, PicTar, miRDB, microT, and miRTarBase. To visualize the data, the Venn diagrams and the Chord diagram were also plotted by https://www.bioinformatics.com.cn. The chart of Fig. 3F was plotted by Microsoft Excel. Whole dataset is provided as supplemental material online S1.

### Usage of generative AI and AI-assisted technologies in the writing process

During the preparation of this work the authors used ChatGPT in order to improve language and readability, with caution. After using this tool/service, the authors reviewed and edited the content as needed and take full responsibility for the content of the publication.

### Statistical analysis

The data were analyzed with the statistical analysis software GraphPad Prism 9 (GraphPad Software, Boston, MA, USA). The measurement data are presented as the average means ± standard deviation (SD). The comparison of 2 groups were conducted with unpaired T-test. The comparisons of multiple groups of measurement data were performed ANOVA with Tukey’s test as post-hoc. P < 0.05 indicated the statistical significance of differences.

### Supplementary Information

Supplementary Information 1.

Supplementary Figures.

## Data Availability

All data generated or analyzed during this study are available in accordance with reasonable requests to the corresponding author. RNA-seq dataset was provided as a supplementary material which can be downloaded online. 10.1038/s41598-024-65373-x
