# Supplementary material for: miR-124-3p downregulates EGR1 to suppress ischemia-hypoxia reperfusion injury in human iPS cell-derived cardiomyocytes
Source: Sci Rep. 2024 Jun 27;14:14811. doi: 10.1038/s41598-024-65373-x (PMC11208498; doi:10.1038/s41598-024-65373-x)
Supplement: Supplementary file 2 — Supplementary Figures. [file 41598_2024_65373_MOESM2_ESM.pdf]

<u>Supporting Information</u>

miR-124-3p Downregulates EGR1 to Suppress Ischemia-Hypoxia Reperfusion Injury in Human iPS Cell-Derived Cardiomyocytes

Qiaoke Yang , Kozue Murata, Tadashi Ikeda, Kenji Minatoya, Hidetoshi Masumoto

Supplementary Figures

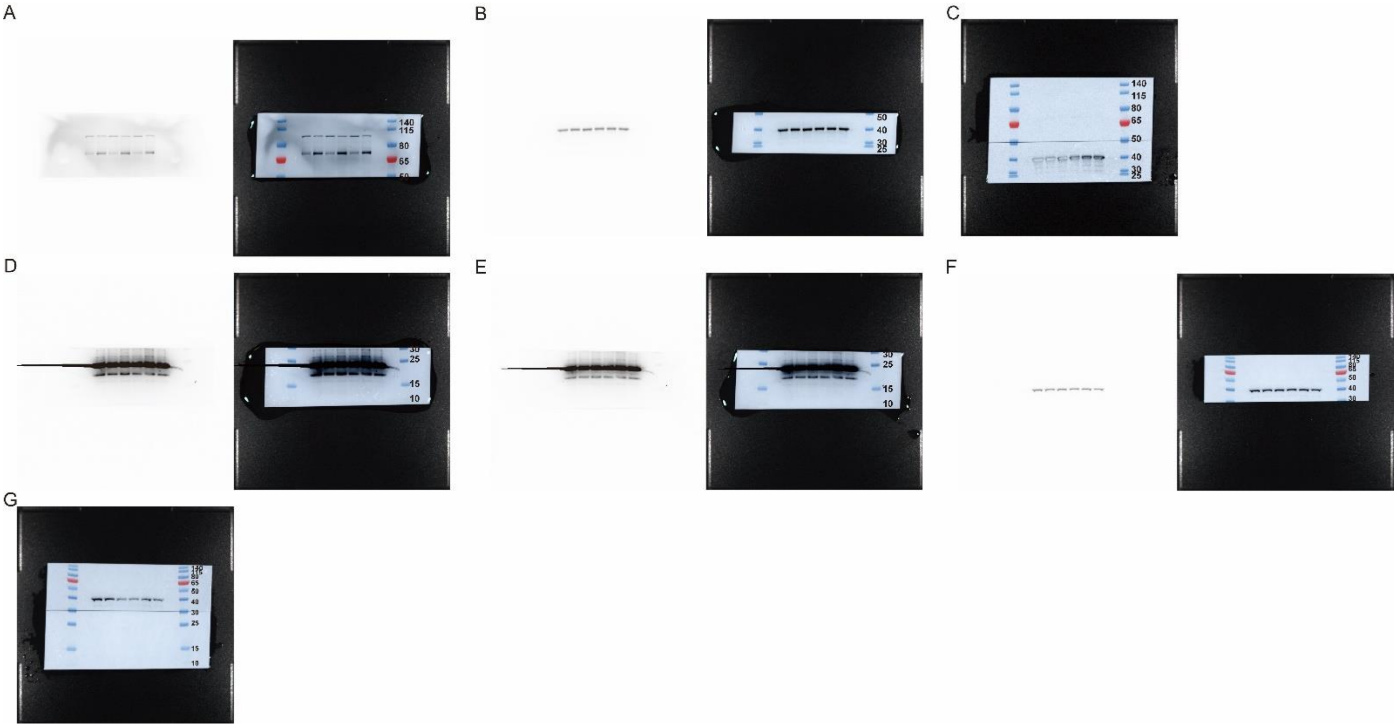

Supplementary Figure 1: The raw images of WB in Fig. 1D, H.

The samples sequence is Control, IH/R, Control, IH/R, Control, IH/R.

(**A-C**) Images for Fig. 1D. (**A**) The raw WB image of EGR1 (75kDa). (**B**) The raw WB image of GAPDH (37kDa). (**C**) The whole membrane image of Fig. 1D. (**D-G**) Images for Fig. 1H. (**D**) The raw WB image of BCL2 (26kDa). (**E**) The raw WB image of BAX (20kDa). (**F**) The raw WB image of GAPDH (37kDa). (**G**) The whole membrane image of Fig. 1H.

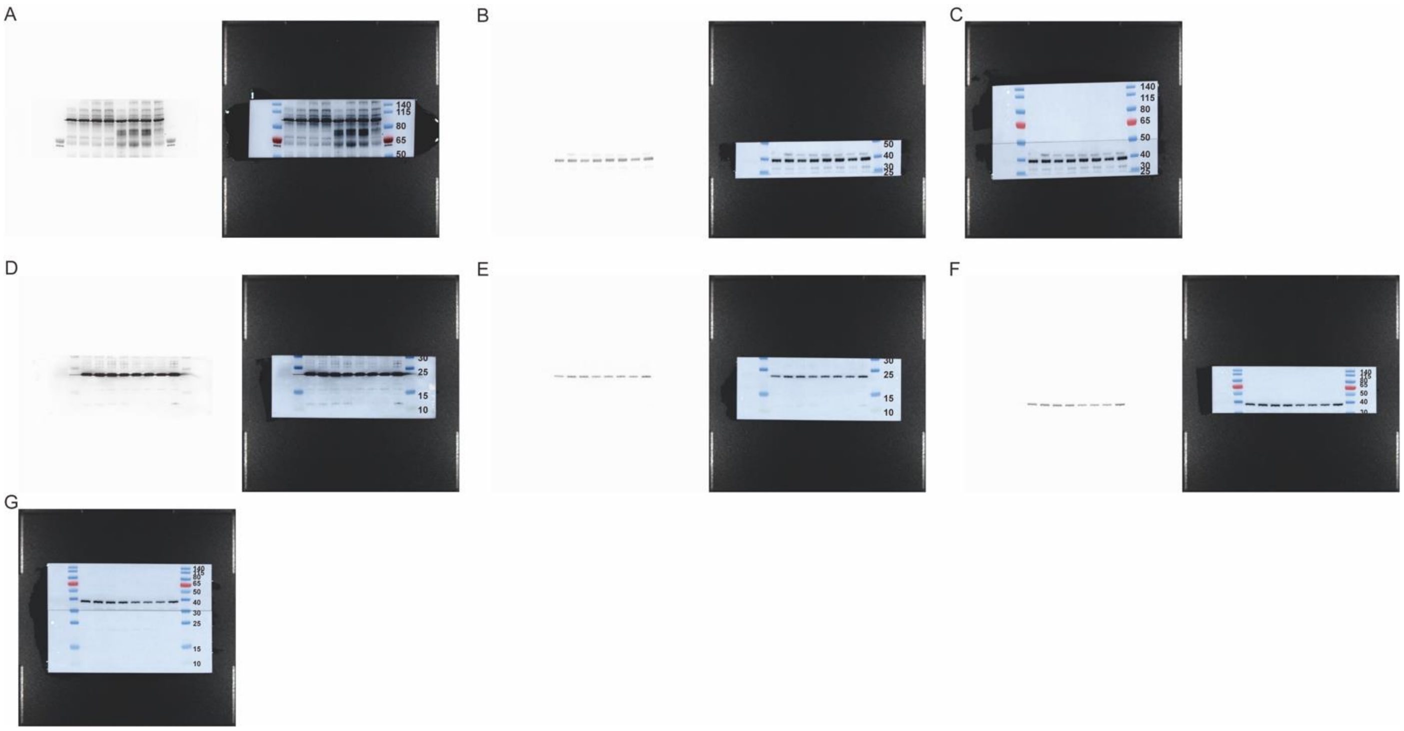

Supplementary Figure 2: The raw images of WB in Fig. 4D, F.

The samples sequence is Control Water, Control Negative miRNA, Control miRNA Positive Control, Control miR-124-3p mimic, IH/R Water, IH/R Negative miRNA, IH/R miRNA Positive Control, IH/R miR-124-3p mimic.

(**A-C**) Images for Fig. 4D. (**A**) The raw WB image of EGR1 (75kDa). (**B**) The raw WB image of GAPDH (37kDa). (**C**) The whole membrane image of Fig. 4D. (**D-G**) Images for Fig. 4F. (**D**) The raw WB image of BCL2 (26kDa). (**E**) The raw WB image of BAX (20kDa). (**F**) The raw WB image of GAPDH (37kDa). (**G**) The whole membrane image of Fig. 4F.
